# Regulatory mechanisms and multidimensional applications of blueberry anthocyanins: a review of current gaps and future directions

**DOI:** 10.48130/els-0026-0005

**Published:** 2026-07-22

**Authors:** Lingyan Cui, Yingfen Yang, Li Li, Shuai Dong, Libo Liu, Abdul Ghafoor, Qin Yang, Waqar Ahmed

**Affiliations:** 1 College of Life and Health Science, Kaili University, Kaili, Guizhou 556000, China; 2 Provincial Famous Teacher Yang Qin Studio, Kaili University, Kaili, Guizhou 556000, China; 3 Guizhou Key Laboratory of Molecular Breeding for Characteristic Horticultural Crops, Kaili, Guizhou 556000, China; 4 Academy of Science and Technology, Chuxiong Normal University, Chuxiong, Yunnan 675000, China; 5 Qiannan Academy of Agricultural Sciences, Duyun, Guizhou 558000, China; 6 Water and Environmental Studies Centre, King Faisal University, Al-Ahsa 31982, Saudi Arabia; 7 School of Breeding and Multiplication (Sanya Institute of Breeding and Multiplication), School of Tropical Agriculture and Forestry, Hainan University, Sanya, Hainan 572025, China

**Keywords:** Blueberry, Anthocyanin biosynthesis, Transcription factors, Environmental regulation, Health benefits, Metabolic engineering

## Abstract

Blueberries are among the richest fruit sources of anthocyanins, which contribute significantly to fruit quality, flavor, and plant physiology. Anthocyanins also exhibit various health-promoting effects, making them valuable in the food, pharmaceutical, and cosmetic industries. Although the structures of anthocyanins and the general framework of their biosynthesis have been largely elucidated, the precise regulatory mechanisms governing anthocyanin production in blueberries remain incompletely understood. Here, we analyze recent advances in understanding blueberry anthocyanin regulation at the enzymatic pathway level (*CHS, CHI, F3H, DFR, ANS*) and the genetic level (*MYB, bHLH, WD40* transcription factors), with particular emphasis on the roles of environmental factors such as light and temperature. This review also explores the diverse health benefits of blueberry anthocyanins, including their antioxidant properties and potential role in preventing chronic diseases such as cardiovascular disorders, neurodegeneration, and cancer. Furthermore, we discuss practical applications of anthocyanins in food, cosmetics, textiles, and pharmaceuticals, as well as emerging uses in smart packaging. Importantly, we highlight how metabolic engineering, synthetic biology, and bioprocess engineering strategies can be leveraged to enhance anthocyanin biosynthesis, stability, and yield in blueberry-based systems. By integrating insights into biosynthetic mechanisms, health impacts, and practical applications, this review provides a comprehensive framework for future research to maximize the potential of blueberry anthocyanins.

## Introduction

Blueberry (*Vaccinium* spp.) is a perennial shrub, a popular fruit, and a source of bioactive compounds worldwide^[1]^. Blueberries are considered a superfood and have antioxidant, anti-inflammatory, and anticancer properties. Previous studies have shown that their active ingredients reduce the risk of cardiovascular disease^[2]^, slow aging, and improve vision and memory. Among these, anthocyanins are recognized as the principal functional compounds responsible for these health benefits^[3]^. Blueberries contain some of the highest levels of anthocyanins (up to 4.95 mg g^−1^) and are regarded as one of the best food sources for these compounds^[4]^. Anthocyanins are natural, water-soluble polyphenols that are primarily stored in the epidermis of fully mature fruit and are important for fruit quality and coloration^[5]^. They offer a wide range of health-promoting benefits, including strong antioxidant, anti-inflammatory, neuroprotective, and vision-enhancing properties^[6]^. In recent years, substantial research has focused on anthocyanin metabolism. Key structural genes involved in the anthocyanin biosynthetic pathway, such as *CHS*, *F3H*, *DFR*, *ANS*, *PAL*, and *CHI*, have been isolated and functionally characterized. The regulation of this pathway is largely mediated by transcription factors, specifically the MBW complex comprising *R2R3-MYB*, basic helix-loop-helix (*bHLH*), and WD40-repeat proteins. These regulatory factors bind to the promoters of structural genes, either activating or repressing their expression in a tissue-specific manner^[7]^.

Anthocyanins are closely associated with the unique flavor and nutritional properties of blueberries and are also involved in fruit color formation during ripening. Therefore, the molecular and cellular processes underlying blueberry color have been a major focus of scientific research^[8]^. Blueberries are rich in the anthocyanin glycosides delphinidin, cyanidin, malvidin, petunidin, and peonidin. Several high-quality blueberry genomes have recently been released, providing a valuable resource for molecular biological research^[9]^. For instance, expressed sequence tag (EST) analyses of ripening fruit have profiled the expression of flavonoid-related genes. Among these, *ANS*/*LDOX* and *UFGT* are highly expressed in red, ripe fruit, and are strongly associated with anthocyanin accumulation during ripening^[10]^. Furthermore, the expression of *ANS* and *DFR* is rapid during mid-ripening. The signaling pathways and key transcription factors for anthocyanin accumulation have recently been characterized, providing targets for genetic engineering and breeding programs. These advances not only enhance the appearance and utility of blueberries but also make them more appealing to health-conscious consumers^[11]^. The importance of blueberry anthocyanin content has attracted significant attention in nutrition and biomedical research, reflecting global interest in natural remedies and functional foods. Understanding the regulatory mechanisms of anthocyanin biosynthesis will facilitate improvements in fruit nutritional quality and support the development of biofortified blueberry cultivars with higher anthocyanin content, helping address nutritional problems.

Given its exceptional anthocyanin content and the availability of high-quality genomic resources, blueberry is an ideal model for investigating the molecular regulation of anthocyanin biosynthesis. Recent advances in sequencing technologies and functional genomics have accelerated the identification of key structural genes and regulatory networks governing this pathway^[12]^. However, fundamental questions remain: How are diverse environmental signals, including light quality, temperature, and phytohormone status, integrated at the transcriptional level to fine-tune anthocyanin output? What epigenetic mechanisms underlie the striking cultivar-specific variation in anthocyanin profiles? This comprehensive review synthesizes current scientific knowledge on blueberry anthocyanins, providing an integrated analysis of their biosynthesis, health benefits, and practical applications. Specifically, this review seeks to: (1) elucidate the complex genetic and enzymatic regulatory mechanisms governing anthocyanin production, including the roles of key structural genes and transcription factors, as well as the influence of environmental and agronomic factors such as light and temperature; (2) document the substantial evidence supporting their multifaceted health-promoting properties, including antioxidant, anti-inflammatory, neuroprotective, and anticancer effects; and (3) explore the diverse industrial applications of these compounds in functional foods, nutraceuticals, cosmetics, and innovative packaging, while also identifying existing challenges related to stability and bioavailability and outlining future research directions to maximize their potential.

## Anthocyanins: structure, properties, and distribution

Anthocyanins are a diverse group of water-soluble pigments in the flavonoid family. They produce a wide range of plant colors and play crucial roles in plant physiology and human health. Understanding their structure, characteristics, and distribution is essential to elucidating their functions and potential applications.

### Chemical structure and types of anthocyanin in blueberry

**Figure 1:**
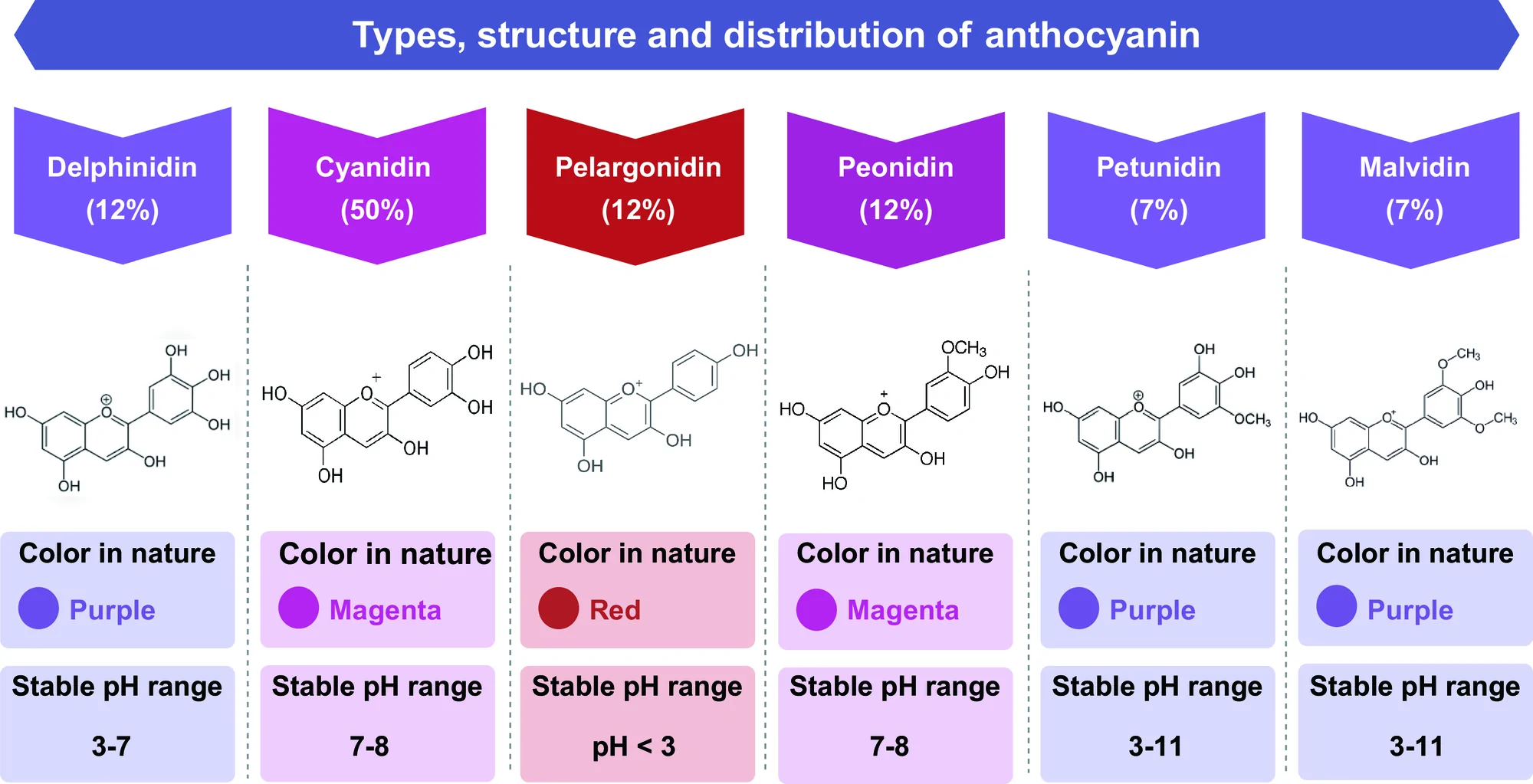
Image depicting the six basic chemical structures, colors, and the distribution of anthocyanins.

Anthocyanins are derivatives of the flavylium cation, characterized by two benzoyl rings (A and B) and a heterocyclic ring (C). They typically occur as glycosides or diglycosides linked to sugars such as glucose, galactose, arabinose, rhamnose, or uronic acid^[13]^. Figure 1 illustrates the six fundamental anthocyanidin structures found in blueberries. Among these, cyanidin accounts for approximately 50% of total anthocyanidins, delphinidin for 12%, peonidin and pelargonidin for smaller proportions, and malvidin and petunidin each constitute about 7%^[14]^. Anthocyanins are glycosylated forms of anthocyanidins (aglycones). Their structural variability and color diversity depend on several factors, including B-ring substitution patterns, glycosylation, acylation, temperature, co-pigments, and pH. The stability of fruit anthocyanins is further influenced by methoxyl or hydroxyl groups on the B ring^[15]^. Glycosylation enhances water solubility and stability through intermolecular hydrogen bonding, whereas acylation reduces solubility but may improve stability under certain conditions.

### Distribution of anthocyanins in blueberries

The distribution and concentration of anthocyanins in blueberries vary significantly among cultivars. Wang et al. analyzed 62 blueberry cultivars and found that 'Rubel' had the highest anthocyanin content, while 'Puru' had the lowest. Among 74 cultivars, 'Gardenblue' had the highest total anthocyanin concentration^[16]^. Cultivar-specific anthocyanin profiles were also observed: 'Legacy' and 'Gardenblue' displayed similar patterns, whereas 'Brightwell' and 'Misty' differed markedly. Malvidin glycosides dominated across genotypes, ranging from 585.4 to 2,556.4 mg kg^−1^. Malvidin accounted for 21.4%–43.7% of total anthocyanin content, depending on the cultivar, while delphinidin comprised 20.8%–56.6%, although it is generally less abundant than malvidin. In some lowbush and highbush varieties, delphinidin has been reported as the predominant anthocyanin^[17]^. Petunidin accounted for 7.8%–20.4% of total anthocyanins, with higher levels observed in half-high, southern highbush, and lowbush blueberries than in cyanidin^[18]^. Cyanidin contributed 4.2%–18.2% of total anthocyanins, while peonidin was the least abundant, ranging from 0.6% to 3.8%, with higher concentrations found in rabbiteye blueberries^[18]^. These findings highlight substantial inter-cultivar variability not only in total anthocyanin content but also in the relative distribution of individual anthocyanin derivatives.

Differences in anthocyanin levels among blueberry cultivars are driven by many interacting factors, including cultivar, environmental conditions, fruit maturity, growing practices, and analytical techniques. A major factor is genetic variation, as research indicates that cultivars and ecotypes (highbush and lowbush blueberries) have distinct anthocyanin profiles and antioxidant capacities^[19]^. Environmental variables such as altitude and temperature are also important; for example, although blueberries grown at lower altitudes tend to be lower in anthocyanin content, higher altitudes can compensate for this lower initial content by increasing anthocyanin accumulation in the later stages of ripening^[20]^. Fruit maturity is also a key factor, as anthocyanin levels increase with fruit maturation, peaking when most fruit is ripe^[21]^. Despite these findings, current comparative investigations have some drawbacks, including the use of different methodologies across studies, making it difficult to compare anthocyanin levels across investigations. In addition, the high genotype × environment interactions observed indicate that findings from single studies may not be generalizable, and that more thorough and standardized studies will provide a better understanding of and potential for exploiting the genetic and environmental factors affecting anthocyanin content in blueberries^[22]^. The structures and occurrence of anthocyanins in blueberry fruit, along with their structural diversity, affect their color properties as well as their chemical stability and biological activities. Anthocyanins' susceptibility to environmental stresses is influenced by variations in glycosylation, hydroxylation, and acylation. Therefore, understanding the structural characteristics of anthocyanins is fundamental to understanding the factors associated with anthocyanin stability and bioavailability.

## Factors affecting the stability and bioavailability of anthocyanins

**Figure 2:**
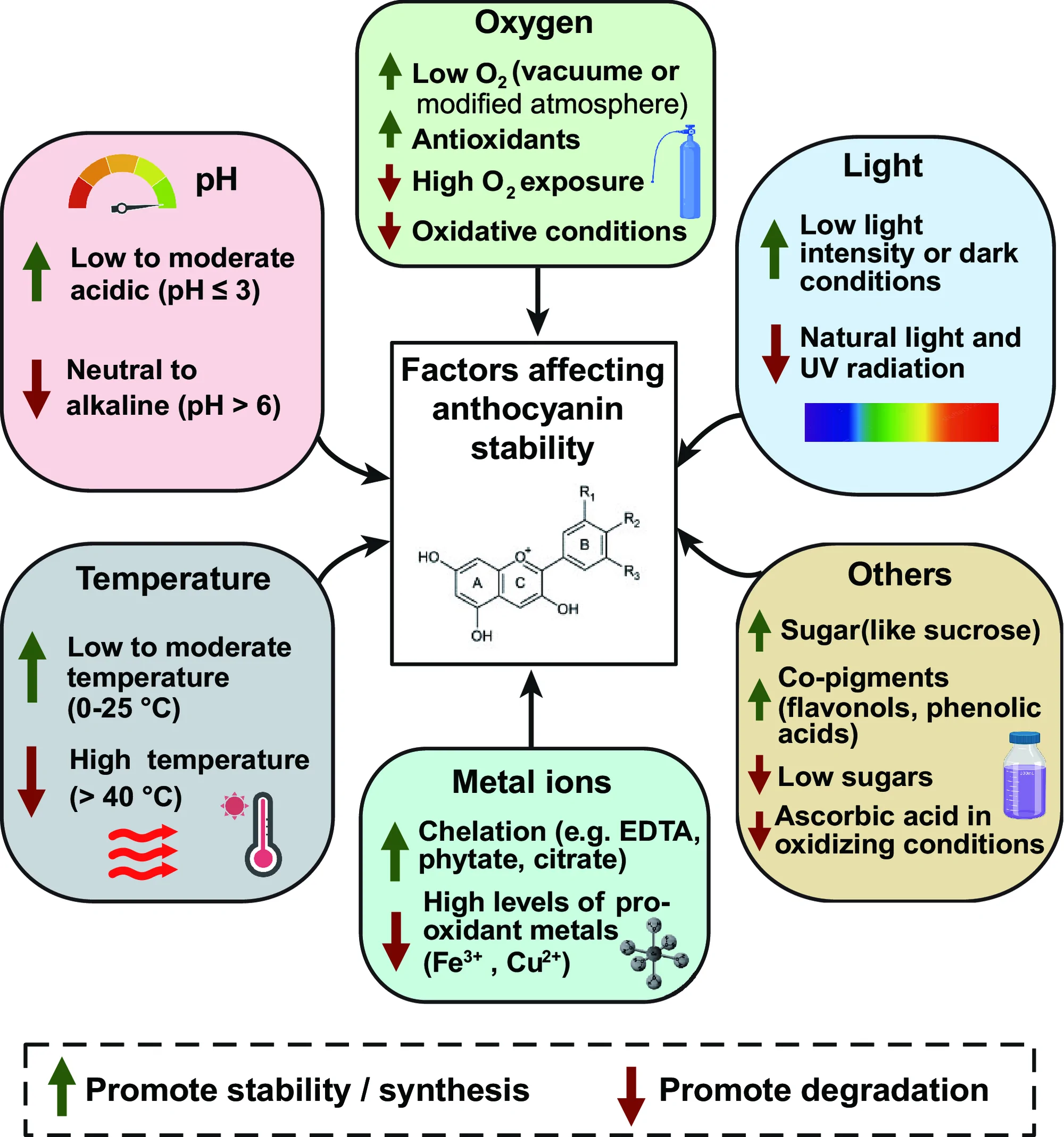
A diagram illustrates the key dynamics affecting the stability and degradation of anthocyanins.

Anthocyanins are flavylium-based molecules with structure-sensitive properties, so their stability and bioavailability are highly sensitive to both basic chemical properties and external environmental conditions. The molecular structure, which can be modified by glycosylation or acylation to enhance stability, is subject to biological activity and degradation at a higher rate under unfavorable environmental conditions. Stability of anthocyanins is thus a key link between their chemical structure and their biological activity in plants and human health. Several environmental and chemical factors (Fig. 2) affect the stability and degradation of anthocyanins. Cardeñosa et al. demonstrated that phenolic compounds in blueberries, particularly anthocyanins, are significantly affected by growing conditions^[23]^. Anthocyanins are unstable and can degrade in response to pH, temperature, ionic strength, light, oxygen, and metal ions, resulting in decreased bioactivity, altered chromatic properties, and reduced bioavailability^[24]^. Anthocyanins are stable in acidic aqueous solutions (pH 2–3) but become increasingly unstable at neutral to alkaline pH (6–8), leading to enhanced degradation and consequently reduced bioavailability. The stability of specific anthocyanins declines progressively as pH increases^[25]^. This pH sensitivity is believed to be due to the pyrylium ring, which is easily opened to a chalcone form, thereby allowing anthocyanin degradation.

Similarly, high temperatures during food processing and storage promote the degradation of anthocyanins. In oxygen-rich environments, degradation is accompanied by the formation of brown degradation products associated with thermal treatment. Studies have shown that anthocyanin degradation is an endothermic reaction, primarily controlled by temperature^[26]^. As temperature increases, the degradation rate increases and the half-life decreases. Preserving the structural integrity of blueberry anthocyanins is more conducive at lower temperatures (2–4 °C). Anthocyanin biosynthesis can be stimulated by light, as can the stability of anthocyanins. Plants have been shown to respond to blue light, which can upregulate anthocyanin biosynthesis genes, leading to increased anthocyanin content in blueberries^[27]^. On the other hand, ultraviolet (UV) radiation significantly reduces anthocyanin content, thereby shortening its half-life. Anthocyanin molecules contain unsaturated bonds that make them highly reactive with molecular oxygen. The stability and degradation of anthocyanins are among the most important factors influenced by oxygen. Research has shown that the degradation of anthocyanins in blueberries is faster in aerobic environments than in anaerobic ones^[28]^. The ability of anthocyanins to chelate metal ions, which depends on the type, concentration, and structural characteristics of the metal ion, plays an important role in plant biology and has significant implications for anthocyanin color stability. Fe^3+^ and Sn^2+^ exert stronger effects on anthocyanin stability than Ca^2+^ and Al^3+[29]^. Metal ion concentration is therefore a critical factor influencing anthocyanin stability.

Further, anthocyanin stability in blueberries can be substantially improved through engineering approaches such as encapsulation, complexation, and copigmentation. Encapsulation technologies, including microencapsulation and nanoencapsulation, protect anthocyanins from environmental stresses, including temperature, pH fluctuations, and light exposure. For example, spray-drying microencapsulation using *β*-cyclodextrin, whey protein, and arabic gum has demonstrated high encapsulation efficiency and enhanced anthocyanin stability during storage and processing^[30]^. Likewise, nanoemulsion-based systems improve anthocyanin stability at room temperature and show potential for cosmetic applications. Complexation with polysaccharides such as pectin further enhances anthocyanin stability and bioavailability by increasing resistance to gastrointestinal digestion and thermal degradation^[31]^. Additionally, ternary covalent complexes comprising whey protein isolate, gellan gum, and tea polyphenols improve both thermal stability and antioxidant activity. Copigmentation with phenolic compounds and metal ions further protects anthocyanins from degradation^[32]^, while combining copigmentation and encapsulation may provide synergistic improvements in color intensity and stability^[33]^. Most importantly, the light, temperature, oxygen, and pH conditions that affect anthocyanin stability also control anthocyanin biosynthesis at the molecular level. Anthocyanin synthesis is a dynamic process in plants that is triggered by environmental factors through the coordinated activation of biosynthetic enzymes and transcriptional regulators^[34]^. As such, the biosynthetic and regulatory pathways for anthocyanin accumulation must be investigated to understand how blueberries preserve their anthocyanin levels, stability, and functionality under varying environmental conditions.

## Biosynthesis mechanisms of blueberry anthocyanins

Anthocyanin biosynthesis in blueberries is tightly coordinated by enzymatic, genetic, and environmental regulatory networks that collectively determine anthocyanin composition, accumulation, and stability. Because environmental conditions directly affect both anthocyanin degradation and synthesis, plants employ sophisticated biosynthetic pathways to maintain pigment accumulation and physiological function. Anthocyanin biosynthesis is a well-characterized pathway in plants, in which a specific set of enzymes sequentially converts phenylalanine into anthocyanin pigments. This pathway is a branch of the general flavonoid biosynthetic pathway. Beginning with phenylalanine, it produces 4-coumaroyl-CoA and subsequently generates chalcones, flavanones, dihydroflavonols, anthocyanidins, and finally stable anthocyanins after glycosylation by UDP-glucose flavonoid 3-O-glucosyltransferase (UFGT)^[35]^. Expression of these regulatory genes is influenced by environmental factors, including light quality, temperature, and phytohormones such as jasmonates and abscisic acid (ABA). Understanding these biosynthetic pathways and their regulatory mechanisms is essential for improving anthocyanin content in blueberries, which is desirable for both nutritional and aesthetic qualities.

### Role of the phenylpropanoid and flavonoid pathway

**Figure 3:**
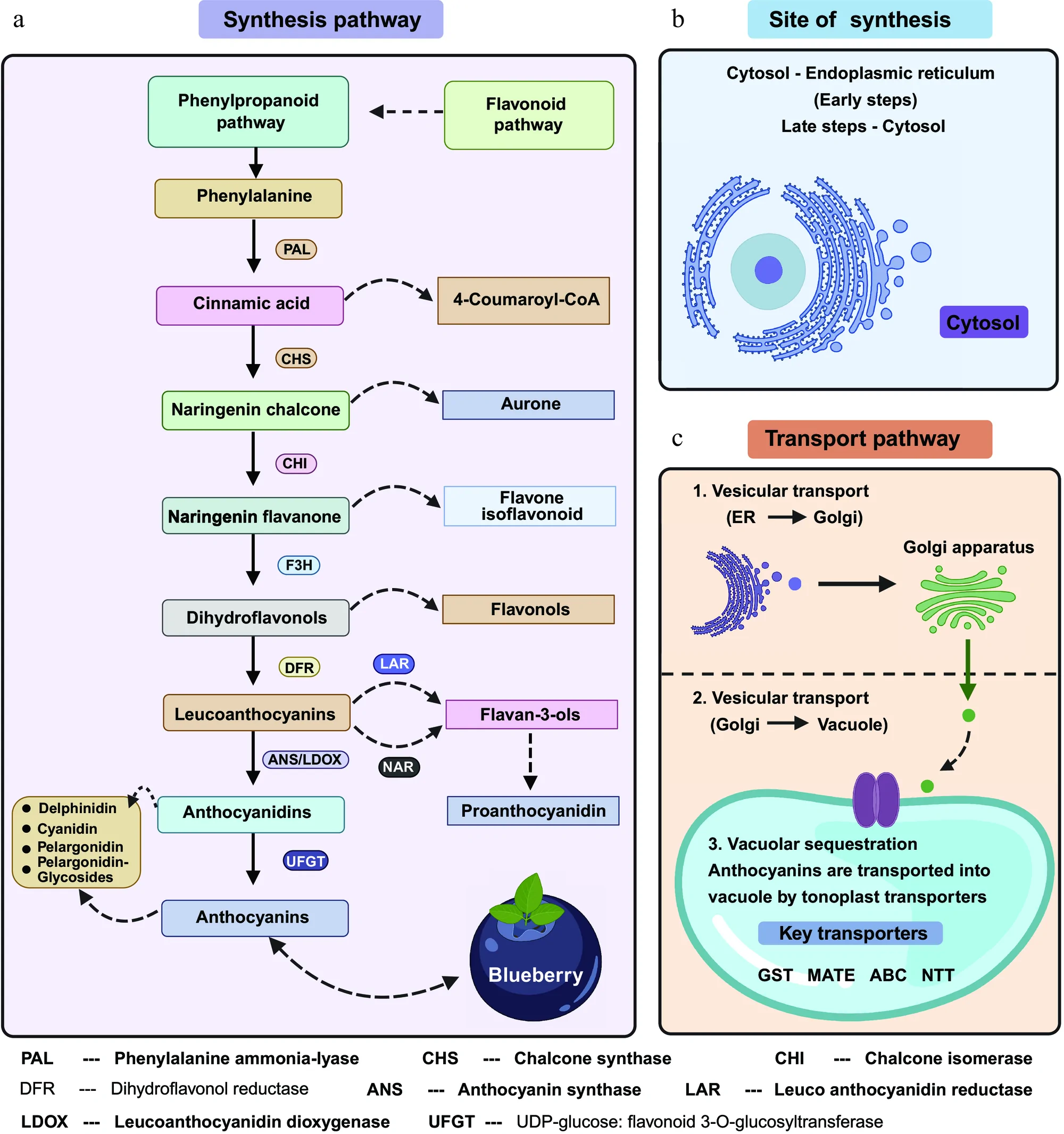
(a) Phenylpropanoid and flavonoid biosynthetic pathways leading to anthocyanin production in blueberry. Phenylalanine is converted through a series of enzymatic reactions catalyzed by phenylalanine ammonia-lyase (*PAL*), cinnamate-4-hydroxylase (*C4H*), and 4-coumarate-CoA ligase (*4CL*) to generate flavonoid precursors. Subsequent reactions involving chalcone synthase (*CHS*), chalcone isomerase (*CHI*), flavanone 3-hydroxylase (*F3H*), flavonoid 3′-hydroxylase (*F3′H*), flavonoid 3′,5′-hydroxylase (*F3′5′H*), dihydroflavonol 4-reductase (*DFR*), anthocyanidin synthase (*ANS*), and UDP-glucose: flavonoid 3-O-glucosyltransferase (*UFGT*) result in the formation and stabilization of anthocyanins. (b) Site of anthocyanin synthesis. (c) Transport pathway of anthocyanins.

The flavonoid biosynthetic pathway, operating through the phenylpropanoid pathway, culminates in the production of anthocyanins and is extensively characterized in the scientific literature (Fig. 3). The specific flavonoid biosynthetic route is initiated by a condensation reaction between one molecule of 4-coumaroyl-CoA and three molecules of malonyl-CoA, catalyzed by chalcone synthase (CHS), yielding naringenin chalcone. Subsequently, the pathway branches into pathways that synthesize various flavonoids, including anthocyanins. The flux can be directed toward the production of delphinidin and cyanidin anthocyanidins through the actions of flavanone 3-hydroxylase (F3H) and flavonoid 3'5'-hydroxylase (F3'5'H). The synthesized anthocyanidins are derived from leucoanthocyanidins via leucoanthocyanidin dioxygenase/anthocyanidin synthase (LDOX/ANS), then glycosylated by UFGT and methylated by O-methyltransferases^[36]^, which catalyze the synthesis of O-methylated anthocyanins such as malvidin, petunidin, and peonidin. Most enzymes of the flavonoid biosynthetic pathway are cytosolic. After biosynthesis, flavonoids are transported to the vacuole or cell wall. Some studies suggest that flavonoid biosynthetic enzymes may function as a metabolon, acting in concert to influence the pathway's overall efficiency, specificity, and regulatory mechanisms^[37]^. Moreover, the overall stability of the biosynthetic pathway is affected by the individual efficiency of each enzyme.

### Molecular regulation of anthocyanin biosynthesis

The anthocyanin biosynthetic pathway comprises six structural genes, grouped into two categories. Upstream genes (*CHI*, *CHS*, *F3H*) produce precursors for major flavonoids, whereas downstream genes (*DFR*, *ANS*, *UFGT*) are specifically involved in anthocyanin synthesis^[38]^. The R2R3-MYB and bHLH transcription factors, together with WD40 proteins, regulate the transcription of both early and late biosynthetic genes^[38]^. Environmental cues, such as blue or red/blue light, upregulate *UFGT* and *LDOX*, promoting anthocyanin accumulation^[39]^. Exogenous ABA applied at the late green stage significantly increases anthocyanin levels and upregulates both structural and regulatory genes. This confirms the importance of the MYB–bHLH–WD40 (MBW) complex in controlling anthocyanin accumulation^[40]^. Co-activation by the MYBA and MYBPA subgroups of R2R3-MYB transcription factors has been demonstrated, with *VmMYBA1* and *VmMYBPA1.1* showing preferential activity during berry ripening and under ABA treatment^[41]^. The VcUVR8–VcCOP1–VcHY5 pathway promotes anthocyanin accumulation by upregulating *VcMYB114* and *VcMYBA2* in response to UV-B radiation^[42]^. Additionally, the bHLH protein interacts with WD40 and MYB transcription factors within the MBW complex to modulate anthocyanin biosynthesis, resulting in high expression of *DFR* and *ANS*^[43]^. The importance of this complex is evident in how MYB transcription factors regulate the pathway through protein interactions and responses to environmental signals.

### Transcriptional or genetic regulation of anthocyanin biosynthesis

Transcription factors that activate flavonoid pathway genes have been identified. These genes work together under the control of R2R3-MYB proteins, which interact with bHLH transcription factors and WD40-repeat proteins^[44]^. The expression profiles and DNA-binding specificities of MYB and bHLH proteins together determine which genes are activated, while WD40 proteins perform a more general regulatory function^[45]^. Plant MYB proteins are pivotal in regulating signal transduction, secondary metabolism, developmental processes, and disease resistance^[46]^. MYB genes possess a conserved DNA-binding domain of 100–160 bp. R2R3-MYB genes linked to the flavonoid pathway contain two repeats and constitute the most prevalent MYB class in plants^[47]^. Some R2R3-MYB members uniquely control specific branches of the flavonoid pathway, including anthocyanins, proanthocyanins, and flavonols^[48]^.

MYB transcription factors, particularly R2R3-MYB, control anthocyanin biosynthesis and pigmentation of fruit skin, flesh, and foliage^[49]^. Anthocyanin pathways are activated by an MYB–bHLH–WD40 complex, as in other plants. Blueberries possess an MYBA ortholog and an anthocyanin-inducing R2R3-MYB, suggesting that MYB transcription factors play a general role in anthocyanin regulation rather than specifying allelic variants^[50]^. Research on MYBA and MYB10 in blueberries reveals a complex regulatory network. MYBA belongs to subgroup 6 of the R2R3-MYB family and is essential for inducing anthocyanin production. It restores anthocyanin production in mutant lines and activates synthesis in model plants, demonstrating functional conservation across species^[50]^. While MYB4a acts as a repressor, UV-B irradiation activates anthocyanin biosynthesis mediated by MYB114 and MYBA through the VcUVR8–VcCOP1–VcHY5 pathway. MYBA and MYBPA together control biosynthesis, with MYBA1 and MYBPA1.1 activating *DFR* and *ANS* while differentially regulating *UFGT* and *F3'5'H*^[51]^. The coordinated action of MYBA1 with MYBC2.1-like repressors establishes a regulatory hierarchy in which MYBA1 serves as the central activator^[52]^. Transcriptomic studies confirm MYBA as a major factor in fruit color differentiation and in regulating multiple structural genes. Additionally, exogenous ABA enhances MYBA activity, thereby increasing anthocyanin content during ripening, suggesting its involvement in ABA signaling^[39]^.

### Epigenetic regulation of anthocyanin biosynthesis

Epigenetic regulation of anthocyanin biosynthesis involves histone modifications, DNA methylation, and transcription factor activity. In *Arabidopsis*, an antagonistic interaction between the histone variant H2A.Z and trimethylation of lysine 4 on histone H3 (H3K4me3) influences anthocyanin biosynthesis: H2A.Z represses gene expression and reduces anthocyanin accumulation, whereas H3K4me3 promotes it^[53]^. DNA methylation also plays a critical role during blueberry fruit ripening, as changes in methylation levels correlate with anthocyanin production. For instance, the methylation balance at the promoters of *VcANS* and *VcCHS* is essential for regulating gene expression during this process^[54]^. Additionally, the miR156/SPL12 module regulates anthocyanin biosynthesis by controlling ethylene production, a major signal in fruit color change^[55]^.

Further, DNA methylation and histone modifications play crucial roles in regulating anthocyanin-related genes during blueberry fruit development and coloration. DNA methylation in promoters, especially those of genes involved in anthocyanin biosynthesis (e.g., *VcCHS* and *VcANS*), is closely correlated with anthocyanin accumulation during fruit ripening. The ratio of methylation to demethylation affects the expression of these genes and thus the concentration of anthocyanins in the fruit^[54]^. Further, the contexts do not specifically mention histone modifications, but they do note that histone modifications regulate gene expression and interact with DNA methylation. Multiple transcription factors, such as MYB, bHLH, and WD40, also regulate the expression of anthocyanin biosynthetic genes and form complexes that activate or repress the anthocyanin biosynthesis pathway^[40]^. These transcription factors and their target genes can be influenced by environmental and developmental factors, causing differential anthocyanin accumulation and fruit coloration in different blueberry varieties^[56]^. Besides, potassium has been found to increase anthocyanin production by upregulating the genes that make up the anthocyanin biosynthesis pathway, including the F3H and UFGT genes, and the enzymes involved in the pathway^[57]^. In summary, the interplay among DNA methylation, histone modifications, and the activity of transcription factors creates a complex regulatory network that governs the expression of anthocyanin-related genes in blueberry fruit, which in turn affects their color and quality.

### Environmental and agronomic regulation of anthocyanin biosynthesis

Light is a critical regulator of anthocyanin biosynthesis. Light enhances anthocyanin accumulation in blueberry skin, whereas darkness reduces it. Light quality also influences biosynthesis, with ultraviolet light and specific wavelengths, such as blue light, playing key roles^[27]^. Blue and red light activate structural genes and metabolites in the anthocyanin pathway. Blue light substantially stimulates *LDOX* and *UFGT* expression, leading to anthocyanidin biosynthesis, and induces ABA signaling genes^[51]^. Long-term exposure to visible and near-visible light enhances anthocyanin production via the high irradiance response (HIR), mediated by phytochrome and cryptochrome photoreceptors. Elevated temperatures (30–35 °C) reduce anthocyanin content^[58]^. In *Arabidopsis*, high temperatures inhibit anthocyanin production by promoting the COP1-mediated proteolytic degradation of HY5, thereby altering MYBL2 expression^[59]^. The phenylpropanoid pathway enzymes PAL, CHS, and F3H exhibit variable expression in response to temperature changes. Cold plasma treatment enhances anthocyanin accumulation by upregulating genes in the phenylpropanoid pathway^[60]^. Lower temperatures increase ABA levels and boost expression of anthocyanin biosynthesis genes, illustrating the interaction between temperature and ABA signaling^[61]^.

In addition, numerous recent studies have shown correlations between these environmental variables and changes in the regulatory complex involved in anthocyanin biosynthesis in fruit and have identified stability and regulatory factors involved in anthocyanin biosynthesis across a variety of fruit^[62]^. For instance, a study by Czemmel et al.^[63]^ has shown that some R2R3-MYB genes involved in fruit flavonoid biosynthesis respond differentially to light and other environmental stimuli. Low (15 °C) or high (35 °C) temperatures during the lighting or darkening of grapevine berries showed that even within the anthocyanin biosynthesis pathway, three MYB genes (*VlMYBA1-2, VlMYBA2*, and *VlMYBA1-3*) were highly variable in their expression among the MYB genes examined. Conversely, other MYB genes, such as *MYB5a* and *MYB5b*, within the same pathway, were insensitive to the treatments^[64]^. Anthocyanin concentrations were highest in grapevine berries kept at 15 °C, although qualitative differences among the treatments were also observed. Intriguingly, the highest abscisic acid levels were observed in the same treatment. Although the dark treatment (15 °C) showed relatively high levels, this indicates that ABA may be involved in the temperature-mediated regulation of flavonoid synthesis. Light intensity significantly affects anthocyanin levels. Full light intensity yields 1.76–24.13 times higher anthocyanin content than reduced intensities, accompanied by increased endogenous hormones and enzyme activities^[65]^. Blue and red/blue light induce anthocyanin biosynthesis at the transcriptional level, showing an inverse correlation with photosynthetic activity^[35]^. Reducing sunlight exposure by 90% delays fruit maturation by at least 2 weeks and reduces anthocyanin levels by 70%–80%^[66]^. Anthocyanin biosynthesis in fruit is a complex process, regulated by a network of structural genes and transcription factors, with substantial variation among fruit species (Table 1). Together, these multilayered regulatory mechanisms, spanning enzymatic pathways, transcriptional control, epigenetic modulation, and environmental responsiveness, ultimately determine the quantity and composition of anthocyanins accumulated in blueberry fruit.

**Table 1:** Key enzymes, genes, and regulatory factors involved in anthocyanin biosynthesis in blueberry.

Crop	Main enzyme(s)/ gene(s)	Core functions/reaction	Regulatory factors	Engineering interventions	Ref.
Blueberry	*PAL, CHI, DFR, UFGT*	Converting phenylalanine to anthocyanins drives pigment accumulation.	Induced by light and ABA	Precision light engineering using full-spectrum LEDs and greenhouse light-control systems, coupled with metabolic engineering of JA, ABA, and ethylene-responsive pathways.	[65]
Blueberry	*PAL, CHI, DFR, UFGT*	PAL converts phenylalanine to cinnamic acid; CHI catalyzes isomerization of chalcones to flavanones; DFR reduces dihydroflavonols to leucoanthocyanidins; UFGT catalyzes glycosylation of anthocyanidins.	Strongly regulated by light intensity	Optimization of light intensity through controlled shading or greenhouse systems, combined with stage-specific light management and hormonal regulation.	[75]
Blueberry	*PAL, CHI, F3'5'H (VcF3'5'H4), DFR, UFGT*	Drive late-stage anthocyanin biosynthesis and glycosylation leading to pigment accumulation in fruit skin.	Regulated by light signaling and transcription factors (VcMYB1 and VcbHLH004)	Optimization of light intensity (avoiding shading) through controlled cultivation enhances anthocyanin glycosides by upregulating key enzymes (F3′5′H, VcF3′5′H4) and light-responsive transcription factors (VcMYB1, VcbHLH004).	[76]
Blueberry	*VcDFR*	Flavonoid biosynthetic pathways are regulated at the level of gene expression.	R2R3 MYB transcription factors	Overexpression of the transcription factor VcMYB1 enhances anthocyanin biosynthesis in blueberry by activating VcDFR and related structural genes.	[77]
Blueberry	*VcPAL3, VcDFR, VcF3H-2, *and* VcUFGT*	Activation of the VcUVR8–VcCOP1–VcHY5 signaling pathway, which upregulates positive MYB regulators and enhances expression of anthocyanin structural genes.	Stimulated by Ultraviolet-B	Engineering interventions include UV-B irradiation to activate the VcUVR8–VcCOP1–VcHY5 pathway and upregulate VcMYBA2 and VcMYB114, combined with suppression or editing of VcMYB4a and VcUSP1 to relieve repression and enhance anthocyanin accumulation.	[42]
Blueberry	*PAL, CHI, DFR, UFGT, VcF3'5'H4, VcbHLH004*	The phenylalanine-derived flavonoid pathway produces anthocyanins, with CHI, DFR, and UFGT catalyzing key conversion steps; VcF3'5'H4 promotes delphinidin-3-O-arabinoside accumulation, while VcbHLH004 regulates anthocyanin biosynthetic genes.	Light intensity and transcriptional regulation via VcbHLH004–VcF3'5'H4	Controlled light intensity enhances anthocyanin accumulation by upregulating key genes (VcF3′5′H4 and VcbHLH004), which can be targeted through light management and gene engineering to improve biosynthesis in blueberry.	[78]
Wild bilberry	Catabolic ABA-8'hydroxylase	Supplemental red and blue light enhance anthocyanin accumulation by upregulating structural anthocyanin genes, key MYB transcription factors, and ABA biosynthesis/signaling components.	Promoted by blue or red light	Supplemental red and blue light enhance anthocyanin accumulation in bilberry by upregulating structural genes, MYB regulators, and ABA biosynthesis.	[51]
Blueberry	PAL, CHI, DFR, UFGT	Phenylalanine is converted through flavonoid pathway intermediates into anthocyanins, resulting in pigment accumulation.	Light wavelength-dependent regulation, especially blue and white light.	Engineering interventions involve using wavelength-specific LED lighting to enhance anthocyanin gene expression.	[27]
Blueberry	*VcMIR156a/VcSPL12*	The VcMIR156a/VcSPL12 regulates blueberry fruit color change by modulating ethylene biosynthesis via direct control of VcACS1 and VcACO6, as well as altering anthocyanin.	miR156/SPL12	Genetic manipulation of the miR156/VcSPL12 module together with regulation of ethylene biosynthesis genes (VcACS1, VcACO6) and ethylene treatments can control anthocyanin accumulation.	[79]

### Engineering strategies for enhanced anthocyanin synthesis and metabolism in blueberry

With growing interest in the high-value nutraceutical potential of blueberry anthocyanins, extensive research has focused on improving the biosynthesis, accumulation, stability, and bioavailability of these compounds through engineering. Recent advances in metabolic engineering have enabled the targeted manipulation of anthocyanin biosynthetic pathways in both plants and microbial systems. Much attention has been paid to controlling key structural and regulatory genes involved in anthocyanin biosynthesis. For instance, the expression of transcription factors such as AtPAP1 and ZmLc has been shown to be crucial for upregulating anthocyanin biosynthesis and thus improving fruit pigmentation and health-promoting attributes^[67]^. Likewise, anthocyanin accumulation and the antioxidant capacity of blueberry products have been shown to be influenced by overexpression of the MYB transcription factor^[68]^. These include large-scale metabolic engineering projects aimed at biofortifying food crops by reinforcing specific metabolic pathways to improve nutrition and human health^[69]^. In addition, the use of synthetic biology techniques, such as multigene stacking and pathway engineering, has enabled more precise control of anthocyanin production and phytonutrient accumulation^[70]^.

Bioprocess engineering is also an effective strategy to enhance anthocyanin yield, extraction efficiency, and product quality in blueberry cultivars, alongside genetic and metabolic engineering strategies. Compared with other emerging technologies, Pulsed Electric Field treatment has been shown to be very promising for extracting juice (32% increase in juice yield) and anthocyanins (55% increase in anthocyanin content) without degrading individual anthocyanin compounds, thereby improving extraction efficiency and product quality^[71]^. Anthocyanin accumulation can also be influenced by agronomic interventions: for example, placing reflective films under blueberry plants increases light interception, photosynthetic activity, and organic matter synthesis, leading to better fruit quality and higher anthocyanin levels during fruit development^[72]^. Other technologies are used for extraction and downstream processing to maximize anthocyanin recovery. Optimizing extraction parameters such as enzyme type, temperature, pH, and process duration is important, as enzyme-assisted extraction (EAE) optimized by response surface methodology (RSM) has been reported to increase anthocyanin yield from blueberry wine residues by 20.8%. Likewise, microbial fermentation under high hydrostatic pressure has been reported as a novel method that improves anthocyanin synthesis and extraction efficiency, yielding highly purified anthocyanin fractions for industrial applications at scale^[73]^. Furthermore, high-voltage electric field extraction followed by additional purification steps can yield blueberry extracts with higher anthocyanin content and improve energy efficiency and process productivity^[74]^. These metabolic and bioprocess engineering strategies offer complementary approaches to improve anthocyanin biosynthesis, extraction, and stabilization in blueberry systems. Their integration offers promising opportunities for developing high-anthocyanin blueberry cultivars and efficient production platforms to meet the growing demand for natural antioxidants and functional ingredients across the food, pharmaceutical, and nutraceutical industries.

## Potential health benefits of blueberry anthocyanins

**Figure 4:**
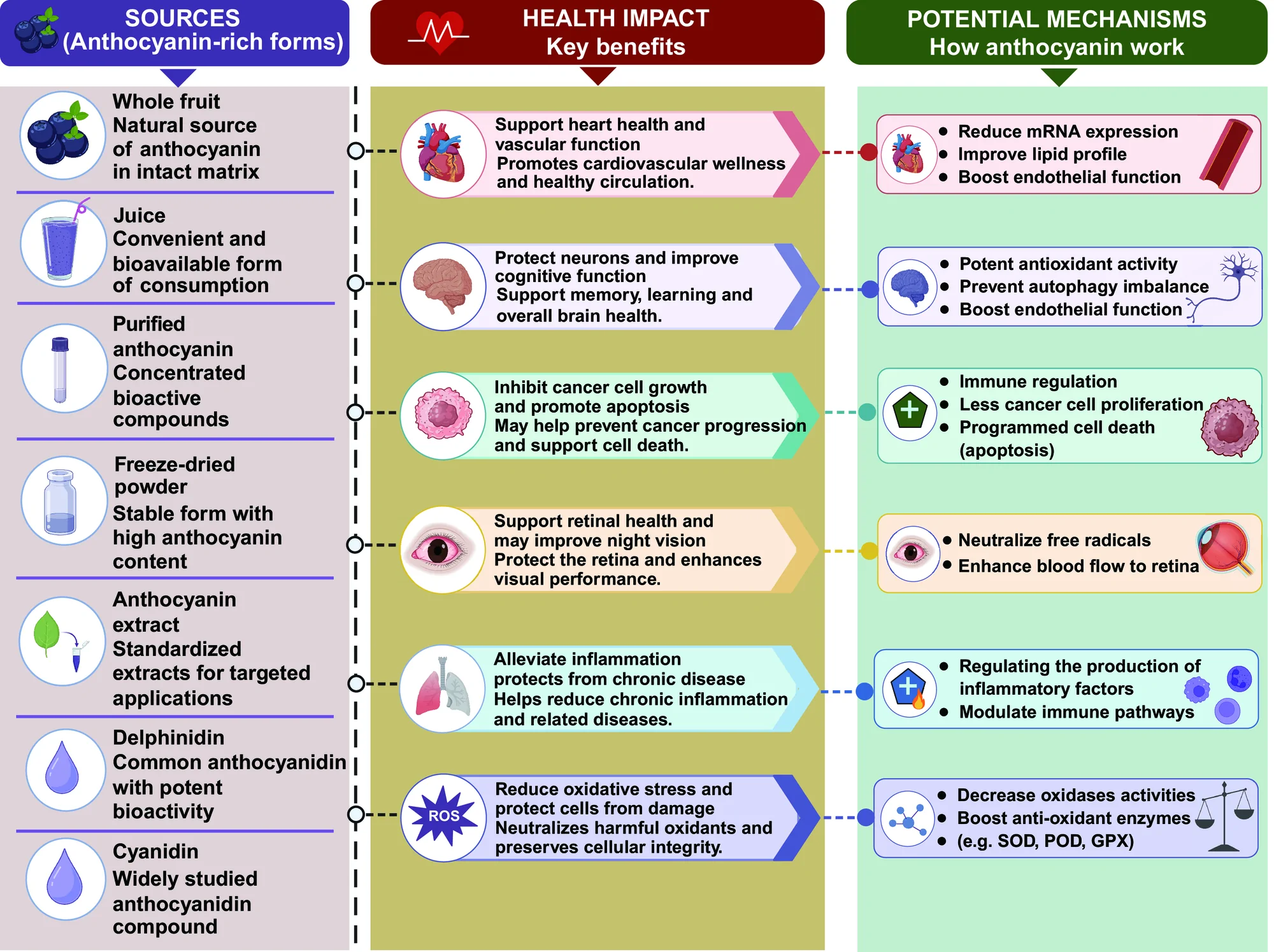
Diagram showcasing the sources of blueberry anthocyanins and their impact on health, indicating potential mechanisms.

Because these biosynthetic and regulatory processes shape anthocyanin composition and abundance, they directly influence the bioactive potential of blueberries, which underpins their diverse health-promoting effects in humans. The anthocyanins found in blueberries have been extensively studied and have been shown to have a wide range of antioxidant, anti-inflammatory, neuroprotective, and anticancer properties. These compounds have been found to be active in biochemical pathways and have the potential to inhibit and protect against several diseases. The key health benefits are outlined in Table 2 and shown in Fig. 4.

**Table 2:** Experimental evidence on the health promoting effects of blueberry anthocyanins.

Health benefits	Treatments/doses	Key findings	Ref.
Antioxidant	Isolation of anthocyanins from blackberry/1 mg mL^−1^	The anthocyanins derived from these berries may significantly mitigate oxidative stress, enhancing their prospective health advantages.	[87]
	Blueberry anthocyanins extract (BAE)	BAE mitigated arsenic-induced reductions in antioxidant capacity in rat hippocampal neurons. It enhanced the expression of proteins associated with mitochondrial biogenesis, essential for cellular health.	[88]
	Anthocyanin extracts of Polaris's blueberry variety/20 μg mL^−1^	Blueberry anthocyanin extracts confer hepatoprotection against acrylamide toxicity. Additionally, they elevate SOD and CAT activities while diminishing MDA levels.	[89]
Anti-inflammatory	BAE	BAE reduced the release of pro-inflammatory cytokines dose-dependently, including monocyte chemo-attractant protein-one, interleukin-6, and cancer necrosis factor-*α* in RAW264.7 cells.	[90]
	Refined and homogeneous extract of bilberry and blackcurrant	Inhibit the TNF-*α*-induced NF-*κ*B pathway in Caco-2 cells, resulting in decreased IL-6 and IL-8 production.	[91]
	0.58 mg mL^−1^ of BAE content incorporated to collagen tempted arthritis rats	BAE exhibited anti-inflammatory characteristics and can reduce osteophyte growth, bone absorption, and soft tissue edema.	[92]
Cardiovascular	160 and 20 g of fresh and freeze-dried blueberry, respectively	Elevated plasma NO_2_ levels do not affect total cholesterol, SBP, HDL-C, LDL-C, DBP, and glucose.	[93]
	Blueberry anthocyanin (20.0 μg mL^−1^)	Substantially improved cellular viability and diminished the rate of apoptosis.	[6]
	Blueberries at 75 and 150 g	Enhanced endothelial function while reducing the level of cyclic guanosine monophosphate and lowering arterial stiffness.	[94]
Neuro-protective	For the first 7 d, BAE was applied at 175 mg kg^−1^, followed by a combination of BAE and acrylamide (35 mg kg^−1^) fed orally for the next 12 d to rats.	BAE lowers MDA levels, increases GSH and antioxidant enzymes, and reduces microglial activation and proinflammatory cytokines. BAE enhances the ERK/CREB/BDNF signaling pathway and reduces A*β* peptide accumulation, protecting neurons and synapses and improving general brain health.	[95]
	Blueberry powder at 269 mg for 24 weeks	This compound is advantageous for the nervous system and aids in memory discrimination prevention.	[96]
	In rats with ketamine-induced hyperactivity, 200 mg kg^−1^ of blueberry extract was given once daily for 14 d.	Blueberry extracts mitigated hyperlocomotion, oxidative stress, and inflammation induced by ketamine.	[97]
Anticancer	Isolation of anthocyanins from blackberry/1 mg mL^−1^	Anthocyanins from Andean blueberry demonstrated significant antitumoral effects in diverse cancer cell lines. This indicates their potential as supplementary or preventive cancer therapies.	[87]
	BAE from five varieties	Anthocyanins may inhibit angiogenesis, a key factor in tumorigenesis. Therefore, blueberries may contribute to cancer prevention via their phytochemical content.	[98]
	BAE (250 µg mL^−1^) and anthocyanin pyruvate adduct were treated in two breast cancer cell lines (MDA-MB-231 and MCF7).	The study found that BAE inhibited cell proliferation in both cell lines.	[99]
Vision improvement	Blueberry anthocyanins (20 mg kg^−1^)	Anthocyanins in blueberries mitigate cognitive deficits.	[100]
	Anthocyanin and polyphenol were 177.8 ± 8.3 and 602.9 ± 9.2 mg 100 g^−1^, respectively.	Blueberry anthocyanins significantly enhance vision by safeguarding retinal cells from photic injury and improving retinal blood flow.	[101]
	For 12 weeks, rats with STZ-induced diabetes were given oral doses of blueberry anthocyanins (20, 40, and 80 mg kg^−1^).	Blueberry anthocyanins mitigate diabetic retinopathy and retinal anomalies.	[102]

### Antioxidation

Anthocyanins are potent antioxidants due to their chemical structure, which allows them to donate hydrogen atoms from the phenolic ring to neutralize free radicals. Their antioxidant capacity is about 50 and 20 times that of vitamin E and vitamin C, respectively, making them promising natural antioxidants^[80]^. Blueberries are rich in anthocyanins and anthocyanidins and are known for antioxidant activity, as shown in laboratory and animal studies^[81]^. Anthocyanins and anthocyanidins account for 84% of the antioxidant potential of blueberry phytochemicals. Anthocyanins in blueberries exhibit strong antioxidant activity, thereby increasing total antioxidant capacity in biological systems^[82]^. Their remarkable reactive oxygen species (ROS) scavenging capacity has been confirmed *in vitro* using ORAC and DPPH assays^[83]^. Human studies have shown that consuming 100 g of freeze-dried blueberry powder, containing 1.2 g of anthocyanins (42% of total phenolic content), significantly increased plasma antioxidant capacity, suggesting that dietary anthocyanins enhance systemic oxidative defense^[84]^.

Blueberry anthocyanin extract reduces oxidative damage in human retinal pigment epithelial cells by lowering malondialdehyde (MDA) and ROS levels while increasing catalase (CAT), superoxide dismutase (SOD), and glutathione peroxidase activities^[85]^. Two principal anthocyanins—malvidin-3-glucoside and malvidin-3-galactoside—significantly reduce ROS and increase SOD in endothelial cells. Antioxidant mechanisms include upregulation of antioxidant enzyme activities and downregulation of oxidase gene expression. Additionally, blueberry anthocyanin extract inhibits acrylamide-induced ROS accumulation, enhances sperm motility, and reduces abnormal sperm count^[86]^. These findings are largely based on *in vitro* and experimental models; therefore, their physiological relevance in humans requires further clinical validation, particularly regarding bioavailability and effective dietary doses.

### Anti-inflammatory effects

Blueberry anthocyanins exhibit pronounced anti-inflammatory effects. They inhibit nitric oxide production in macrophages, a key component of the inflammatory response^[87]^. In inflammatory bowel disease models, blueberry extracts reduced endoplasmic reticulum stress and apoptosis^[103]^. The anti-inflammatory mechanisms involve inhibition of NF-*κ*B translocation and of activator protein, cAMP response element, and CCAAT enhancer binding protein, thereby decreasing NF-*κ*B activity following IL-1*β* stimulation^[104]^. Malvidin-3-glucoside, malvidin-3-galactoside, and their mixtures inhibit TNF-*α*-induced inflammation in a concentration-dependent manner^[105]^. Blueberry extract (0.58 mg mL^−1^ anthocyanin) inhibited the progression of acute inflammation and attenuated clinical symptoms of arthritis, including osteophyte formation, bone resorption, and soft tissue edema in rats with collagen-induced arthritis^[92]^.

### Cardiovascular health

Cardiovascular diseases (CVD) are a leading cause of mortality in advanced economies. Blueberry anthocyanins confer vascular protection by altering lipid metabolism, improving endothelial function, reducing inflammation, and decreasing oxidative stress^[106]^. They also inhibit inflammatory signaling pathways, lowering biomarkers such as C-reactive protein and interleukin-6 linked to cardiovascular risk^[107]^. They activate the PI3K/AKT/eNOS and SIRT1 signaling pathways, enhancing endothelial function and nitric oxide (NO) bioavailability, both of which are crucial for vascular health^[108]^. Blueberry anthocyanins are particularly effective against cholesterol-induced atherosclerosis and ischemic heart disease by lowering blood pressure and mitigating oxidative and inflammatory injury to the endothelium^[109]^. Anthocyanin-rich extracts improve cardiac dysfunction while reducing oxidative stress and inflammation via the DDAH1/ADMA/NO signaling pathway^[110]^. These findings have been confirmed by human studies, as people with high cholesterol who consumed purified blueberry anthocyanins (delphinidin-3-O-*β*-glucoside and cyanidin-3-O-*β*-glucoside) daily lowered inflammatory markers in the blood, including hs-CRP, sVCAM-1, and plasma IL-1*β*. These compounds also lower LDL cholesterol and raise HDL cholesterol levels, thereby improving lipid profiles^[100]^. Most mechanistic evidence comes from preclinical trials; however, long-term randomized controlled trials in humans are still needed to confirm clinical cardiovascular benefits.

### Neuroprotective properties

Blueberry anthocyanins alleviate oxidative stress and mitochondrial dysfunction in neuronal cells, restoring antioxidant capacity and promoting mitochondrial biosynthesis in arsenic-exposed rat hippocampal neurons, thereby improving cognitive function^[111]^. A 12-week wild blueberry juice intervention in nine elderly participants showed gains in neurocognitive performance. Older adults with cognitive decline who consumed 25 g of blueberry powder exhibited increased neural responses to working memory tasks^[112]^. Consuming approximately 148 g of blueberries daily for 16 weeks improved blood oxygen levels in older adults with mild cognitive impairment. Blueberry anthocyanins also mitigate acrylamide-induced neurotoxicity by reducing oxidative stress markers and enhancing ERK/CREB/BDNF neurotrophic signaling pathways, which are crucial for neuronal survival and synaptic plasticity^[95]^. Additionally, they modulate the gut–brain axis, including intestinal microbiota and metabolites, to support brain health^[113]^. Blueberry anthocyanins enhance autophagy and alleviate neuronal damage in Alzheimer's disease models, supporting their role in neurodegenerative conditions^[114]^.

Blueberry anthocyanins have been shown to affect inflammatory signaling pathways *in vitro* and in animal studies. In cellular models, such as RAW 264.7 macrophages, these compounds, often at concentrations exceeding physiological levels (> 10 µM), have been found to inhibit phosphorylation of NF-*κ*B p65 and I*κ*B and to suppress the MAPK p38 and JNK pathways, resulting in decreased production of TNF-*α* and IL-6^[115]^. However, it remains to be confirmed whether these selective pathway inhibitions occur at nanomolar to low micromolar levels of anthocyanin metabolites, which can be found in dietary interventions. The anti-inflammatory activity in humans could be attributed to multiple mechanisms, such as modulation of the gut microbiota and action of more common phenolic acid metabolites^[116]^. Human intervention studies indicate that the consumption of blueberries can have a positive effect on postprandial glucose control and insulin sensitivity, thus lowering oxidative stress and inflammation in the gastrointestinal tract^[117]^. Although the results are promising, there are limited clinical studies of human subjects in this field, and mechanisms observed in animal studies need to be confirmed in well-controlled trials.

### Anticancer potential

By 2030, an estimated 22 million new cancer cases are expected^[118]^. Blueberries, known for their high anthocyanin and polyphenol content, have attracted interest for their potential chemopreventive and anticancer properties, primarily based on promising preclinical evidence^[119]^. Blueberry anthocyanins have been shown to inhibit cancer cell growth, induce apoptosis, modulate immune responses, and inhibit metastasis *in vitro*, *in vivo*, and in clinical studies. In hepatocellular carcinoma cells (HepG-2), blueberry anthocyanins induced ROS generation, disrupted mitochondrial membrane potential, activated caspase-3, and released cytochrome c from mitochondria, leading to apoptosis. These processes are linked to Bcl-2 downregulation and Bax upregulation, both of which suggest activation of the intrinsic apoptosis pathway^[84]^. Blueberry anthocyanins, particularly delphinidin, inhibit cancer cell proliferation and induce apoptosis in several cancer cell lines^[120]^. Blueberry anthocyanins strongly promote HepG-2 cell apoptosis by increasing ROS production, elevating caspase-3 activity and Bax expression, and decreasing mitochondrial membrane potential and Bcl2 expression^[121]^. They also inhibit breast cancer cell viability and induce apoptosis^[99]^, suggesting their potential as natural anti-tumor agents. Anthocyanins extracted from 'Gardenblue' blueberries exert strong anti-proliferative effects against HepG2 liver cancer cells and act synergistically with doxorubicin^[119]^.

Mechanistic studies have revealed that blueberry anthocyanins influence several pathways crucial to cancer development. Cyanidin-3-rutinoside induces ROS-mediated mitochondrial apoptosis in HL-60 leukemia cells^[122]^. *In vitro* studies have demonstrated that blueberry extracts and isolated anthocyanins, often used at concentrations ranging from tens to hundreds of µM, can suppress proliferation and metastasis-related pathways in breast cancer cell lines. These effects are linked to modulation of the PI3K/Akt, MAPK/ERK, and STAT3 pathways^[123]^. A major challenge in translating these findings is that the concentrations used in these studies are much higher than the peak plasma levels of the parent compounds (< 100 nM) observed in humans after consuming blueberries^[124]^. This difference necessitates caution when applying these specific molecular mechanisms to dietary cancer prevention. Direct clinical evidence for the anticancer effects of anthocyanins remains limited, as most findings are based on epidemiological and preclinical studies. Although anthocyanin-rich diets are associated with reduced cancer risk, robust interventional studies confirming therapeutic or preventive effects in humans are still needed.

### Vision improvement

Blueberry anthocyanins show promising potential for improving vision health. Cyanidin-3-glucoside, the major anthocyanin in blueberries, alleviates oxidative stress in retinal cells, which is key to preventing diabetic retinopathy and other retinal abnormalities^[114]^. Berry anthocyanins protect human retinal pigment epithelial cells from light-induced damage^[125]^. In pigmented rabbits, blueberry anthocyanins prevent light-induced retinal damage^[101]^. In diabetic rats, blueberry anthocyanins protect retinal cells from oxidative damage by regulating Nrf2/HO-1 signaling^[102]^. Furthermore, specific anthocyanins like cyanidin-3-O-glucoside have been shown to protect against high-glucose damage via the REDD1/GSK3β pathway, enhancing the antioxidant response and reducing VEGFA expression^[126]^. The protective effects of blueberry anthocyanins against light-induced damage in RPE cells are also observed, with compounds such as cyanidin-3-glucoside demonstrating marked ROS clearance and anti-aging properties^[125]^. A few human intervention studies have examined the effects of blueberry anthocyanins on visual function. In randomized controlled trials, supplementation with cyanidin-3-glucoside equivalents (271–346 mg) for 3–12 weeks showed beneficial effects on visual recovery after retinal photobleaching, with no significant effects on night vision or dark adaptation^[127]^. However, the clinical significance of faster photobleaching recovery in daily vision should be further examined.

## Applications of blueberry anthocyanins

**Figure 5:**
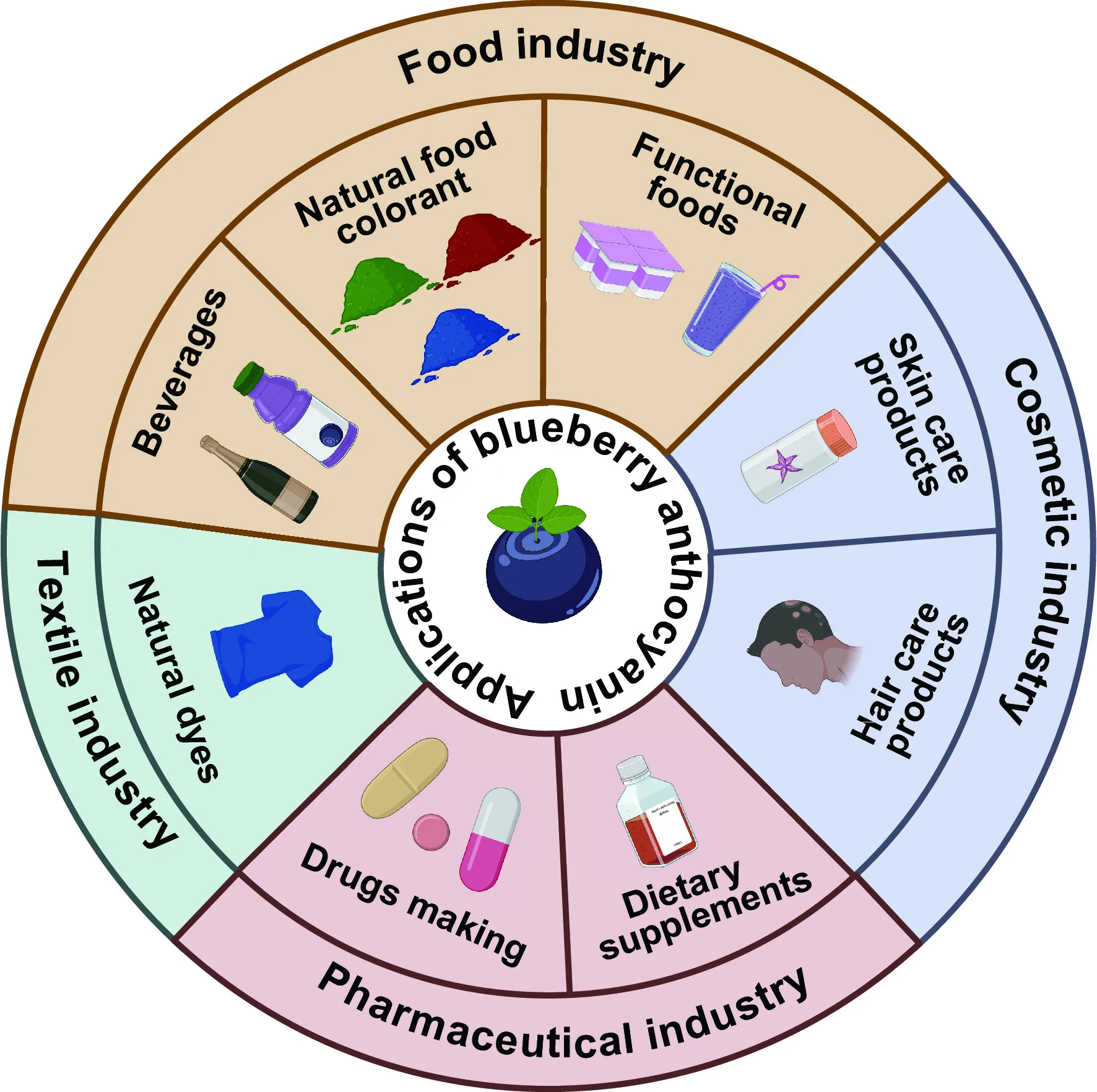
Applications of blueberry anthocyanins across the food, pharmaceutical, cosmetic, and textile industries. The figure highlights the multifunctional roles of anthocyanins and their potential commercial value across diverse industrial sectors.

Blueberry anthocyanins have diverse applications across multiple disciplines due to their varied biological activities and functional characteristics (Fig. 5). Beyond health-related uses, they are used in the food sector as natural colorants, despite challenges related to their inadequate chemical stability and diminished bioavailability^[128]^. Innovative applications have also emerged in renewable energy, where these compounds serve as sensitizers, demonstrating potential technological applications in nations with significant agricultural output. Functional foods and beverages incorporating blueberry anthocyanins are designed to confer health advantages beyond mere nutritional value^[86]^. Such products capitalize on the health benefits of anthocyanins, which are attractive to health-minded consumers. Studies show that including blueberries in the diet can be beneficial for glucose control in sedentary young adults.

According to reports, anthocyanins are best known for their antioxidant and coloring capabilities. A significant use is that they serve as natural color indicators. Anthocyanins extracted from blueberries have been used to dye cotton fibers due to their safety profile and intense color. They are also used in the production of biopolymer-based intelligent packaging films for monitoring food freshness (shrimp and milk). However, dyeing trials have revealed that hydrogen bonding and *π*–*π* interactions between anthocyanins and metallic mordants decrease under optimum dyeing conditions, thus restricting their application in non-food textiles (cotton)^[81]^. Blueberry anthocyanins are widely used in the cosmetics industry. Deep eutectic solvents offer a viable means of stabilizing blueberry anthocyanins for cosmetic applications by preserving their resistance to environmental degradation^[129]^. These solvents enhance anthocyanin stabilization and antioxidant efficacy, which is necessary for their use in skincare formulations^[130]^. This desirable safety profile, combined with their multifunctional benefits, positions blueberry anthocyanins as unique ingredients in the cosmetics industry for natural skin enhancement and protection.

Despite their promising applications, blueberry anthocyanins, like other plant-derived anthocyanins, face significant challenges due to their inherent instability and sensitivity to environmental factors, which complicate their storage, processing, bioavailability, and industrial utilization. These compounds are highly reactive, exhibiting electrophilic, nucleophilic, and electron-donating properties that give them their color variety but also explain their instability to water addition and autoxidation, yielding colorless products^[131]^. This instability is exacerbated by environmental factors such as pH, temperature, light, and oxygen exposure, causing degradation during processing and storage^[132]^. For example, anthocyanins are stable at low pH but are rapidly destroyed at higher pH, higher temperatures, and under light exposure^[28]^. This instability not only affects their color but also their bioactive properties, reducing their antioxidant activity and therapeutic implications.

Beyond stability issues during storage and processing, there are several limitations and deficiencies in the extraction and practical use of blueberry anthocyanins. Traditional extraction methods, frequently relying on thermal technology, can reduce anthocyanin recovery and stability, lowering extraction yields and restricting further use in food systems^[133]^. Innovative extraction technologies, such as pulsed electric field, microwave-assisted, and ultrasound-assisted extraction, have been investigated to improve anthocyanin recovery from agri-food byproducts, but ensuring the stability of these compounds throughout the entire extraction process and during storage has been a challenge^[134]^. In addition, the use of deep eutectic solvents (DESs) in green extraction has demonstrated better extraction efficiency and improved stability of anthocyanins, but the separation and purification of target compounds remain difficult^[135]^. Another significant limitation is the low bioavailability of anthocyanins. However, their absorption, metabolism, and biological fate in the human body are not fully understood, and isolated anthocyanins may not confer the same health benefits as when consumed in whole foods^[136]^. Thus, the instability of anthocyanins during digestion and low systemic bioavailability still limit their application in functional foods, nutraceuticals, and therapeutic applications.

Several stabilization strategies have been proposed to overcome these problems. It is known that the resistance of blueberry anthocyanins to degradation by light and heat can be improved through structural modification. To address these environmental challenges, anthocyanins have been encapsulated using techniques such as microencapsulation and nanoliposome-based delivery systems, which have also resulted in enhanced stability and bioavailability^[137]^. These systems are often based on bio-based materials such as pectin, proteins, and lipids, which help maintain anthocyanin integrity during processing, storage, and digestion and facilitate controlled release^[132]^. Although these developments have been made, it is still unclear how all anthocyanins will behave during processing or what factors contribute to their degradation, and more research is required to fully understand these factors^[138]^. Thus, further research is suggested to optimize extraction techniques, improve anthocyanin stability and bioavailability in blueberries, and develop additional effective delivery systems that would ensure the maximum use of anthocyanins in the health industry and industrial applications. In conclusion, although substantial advances have been achieved in the stabilization of blueberry anthocyanins, additional research is still needed to identify the best methods to optimize them for wider industrial use.

## Conclusions and future perspectives

This review has highlighted significant advances in understanding the regulation of anthocyanin biosynthesis in blueberries, encompassing genetic, metabolic, and environmental interactions. Key transcription factors and regulatory pathways have been identified, providing targets for increasing anthocyanin content through molecular breeding, biotechnological intervention, and optimized agricultural practices. Blueberry anthocyanins are remarkable bioactive compounds with substantial potential to impact human health and various industrial sectors. Their multiple health benefits, including antioxidant, neuroprotective, cardioprotective, anticancer, and anti-inflammatory effects, suggest therapeutic promise. Additionally, the versatility of anthocyanins in functional foods, nutraceuticals, and cosmetic products demonstrates their growing market importance.

Despite these advances, several challenges remain. The instability of anthocyanins under environmental and processing conditions, their poor bioavailability in the human body, and the high costs associated with large-scale production and purification continue to limit their widespread application. To overcome these challenges, future studies should aim to understand the molecular mechanisms that control anthocyanin synthesis, transport, accumulation, and degradation using multi-omics integration approaches. These tools will improve our understanding of the genetic and environmental factors that affect anthocyanin production. The poor bioavailability and stability of blueberry anthocyanins during processing, storage, and digestion are significant hurdles to their therapeutic and industrial use. Thus, it is imperative that advanced delivery systems be developed to enhance stability, absorption, and biological effectiveness, such as nano-encapsulation and biopolymer-based carriers. Another intriguing aspect to explore is the relationship between anthocyanins and the gut microbiota. Research on the bioactivity of anthocyanins in the context of microbial metabolism could help develop individualized nutrition approaches and functional food products.

Additional clinical trials are required to confirm the benefits of blueberry anthocyanins in the prevention and treatment of chronic diseases, including cardiovascular, metabolic, and neurodegenerative diseases. Standardized intervention protocols, along with long-term safety evaluations, will be important for translating laboratory results into clinical practice. On the agricultural side, breeding and genome editing could provide opportunities to develop blueberry cultivars with enhanced yield, nutritional quality, and environmental resilience, while maintaining high anthocyanin content. Furthermore, the potential of blueberry anthocyanins as natural colorants, functional food ingredients, smart packaging indicators, and cosmetics additives will necessitate cost-effective extraction technologies and improved product stability. Overall, interdisciplinary research, combining plant science, food technology, biotechnology, and medicine, will be crucial to realizing the potential health benefits and industrial applications of blueberry anthocyanins.

## Data Availability

Data sharing not applicable to this article as no datasets were generated or analyzed during the current study.
